# Quantum mechanics of 4-derivative theories

**DOI:** 10.1140/epjc/s10052-016-4079-8

**Published:** 2016-04-25

**Authors:** Alberto Salvio, Alessandro Strumia

**Affiliations:** 1 Departamento de Física Teórica, Universidad Autónoma de Madrid and Instituto de Física Teórica IFT-UAM/CSIC, Madrid, Spain; 2 Dipartimento di Fisica dell’Università di Pisa and INFN, Pisa, Italy; 3 Theory Division, CERN, Geneva, Switzerland

## Abstract

A renormalizable theory of gravity is obtained if the dimension-less 4-derivative kinetic term of the graviton, which classically suffers from negative unbounded energy, admits a sensible quantization. We find that a 4-derivative degree of freedom involves a canonical coordinate with unusual time-inversion parity, and that a correspondingly unusual representation must be employed for the relative quantum operator. The resulting theory has positive energy eigenvalues, normalizable wavefunctions, unitary evolution in a negative-norm configuration space. We present a formalism for quantum mechanics with a generic norm.

## Introduction

Newton invented classical mechanics putting two time derivatives in his equation $F=m \ddot{x}$, which corresponds to a kinetic energy with two time derivatives, $m\dot{x}^2/2$. Later Ostrogradski proved a no-go theorem: non-degenerate classical systems with more than two time derivatives contain arbitrarily negative energies and develop fatal run-away instabilities [1]. Classically, they do not make sense.

The discovery that nature is relativistic and quantum opened the quest for an extension of Newtonian gravity. A century ago Einstein and Hilbert found the classical theory of relativistic gravity. However, its quantum version is not renormalizable in 3+1 space-time dimensions. Sticking to the observed number of space-time dimensions, a renormalizable extension of general relativity is found by adding terms quadratic in the curvature tensor to the Einstein–Hilbert Lagrangian, such that the graviton acquires a 4-derivative kinetic term. Stelle proposed and dismissed this extension [2] (see also [3–13]).

Recently the Higgs mass hierarchy problem brought interest to dimension-less theories. In this context, gravitons must have dimension 0 (being a dimension-less metric) and thereby *must* have a 4-derivative kinetic term. If these theories could make sense at the quantum level, despite the negative classical energy, a great deal would be gained: relativistic quantum gravity, plus hierarchies among dynamically generated mass scales [12], plus inflation [14, 15].

Quantization can eliminate arbitrarily negative classical energies. The following example is well known: the classical relativistic spin 1/2 field is described by a spinor $\Psi (x)$ with Dirac Lagrangian  containing one time derivative. Treating $\Psi$ as a classical field (as initially proposed by Schrödinger), and inserting the plane-wave expansion

1 $$\begin{aligned} \Psi (x) = \int \frac{\mathrm{d}^3 p}{(2\pi )^3 \sqrt{2E_p}} [a_{p,s} u_{p,s} \mathrm{e}^{-i p\cdot x} + b^\dagger _{p,s} v_{p,s} \mathrm{e}^{ip\cdot x} ]\nonumber \\ \end{aligned}$$

in the Hamiltonian one finds negative energies in half of the configurations space:^1^

2 $$\begin{aligned}&H = \int \frac{\mathrm{d}^3 p}{(2\pi )^3} \, E_p [a_{p,s}^\dagger a_{p,s} - b_{p,s}b_{p,s}^\dagger ],\nonumber \\&E_p = \sqrt{m^2+\vec p^2} \end{aligned}$$

This classical arbitrarily negative energy is avoided by quantization with anti-commutators, if the vacuum state is appropriately chosen. Indeed, the two-state solution to $\{ b,b^\dagger \}=1$ shows that one can switch annihilation with creation operators by choosing the vacuum to be the state with lower energy.

The spin 0 and spin 1 relativistic fields (described by dimension-1 fields with two derivatives) do not have this issue: the negative-frequency solutions to the Klein–Gordon equation correspond to Hamiltonians with positive energy.

The general lesson is that quantization depends on the number of time derivatives.

The goal of this study is describing if/how systems with four derivatives can be quantized obtaining a consistent theory, in particular of quantum gravity. We will find that a unique structure emerges, which again involves switching annihilation and creation operators.

This will bring us into the territory of negative-norm quanta, avoided like a plague by serious theorists that call them ‘ghosts’, and explored only by notorious crackpots such as Dirac [18], Pauli [19], Heisenberg [20], Pais, Uhlenbeck [21], Lee, Wick [22, 23], Cutkosky [24], Coleman [25], Feynman [26], Gross [27], Hawking [28, 29], ’t Hooft [30, 31] and others, also more recently [32–52]. These works sometimes contain bizarre and confusing statements and obsolete motivations, together with interesting ideas and ad hoc prescriptions.

This paper is structured as follows. In Sect. 2 we review the canonical Ostrogradski formalism. In Sect. 3 we present negative-norm quantum mechanics, the negative-norm harmonic oscillator (Sect. 3.3), and the associated negative-norm representation of a canonical coordinate (Sect. 3.4), with unusual parity under time-inversion *T*. In Sect. 4 we recall that a 4-derivative degree of freedom *q*(*t*) is described by two canonical coordinates: $q_1 = q$ and $q_2 = \dot{q}$. While $q_1$ is *T*-even as usual, $q_2$ is *T*-odd: we argue that thereby it naturally follows the negative-norm representation. The resulting quantum theory is unitary: time evolution preserves the negative norm. The path integral formulation is discussed in Sect. 5. In Sect. 6 we discuss the interacting theory, outline the extension to quantum field theory, and discuss the issue of giving a sensible interpretation to negative norms, via a postulate that generalizes the Born rule. Conclusions are given in Sect. 7.

## The Ostrogradski classical canonical formalism

Let us now introduce the main issues in the simplest relevant case. Our final goal will be 4-derivative gravity; however, the graviton components can be Fourier expanded into modes with given momentum and four time derivatives, and at leading order in the perturbative expansion one has decoupled harmonic oscillators. So, we start considering a single mode *q*(*t*), described by the Lagrangian

$$\begin{aligned} \mathscr {L}= & {} -\frac{\ddot{q}^2}{2} + (\omega _1^2+\omega _2^2) \frac{\dot{q}^2}{2} - \omega _1^2 \omega _2^2 \frac{q^2}{2}-V(q) \nonumber \\= & {} -\frac{1}{2} q \left( \frac{\mathrm{d}^2}{\mathrm{d}t^2} + \omega _1^2\right) \left( \frac{\mathrm{d}^2}{\mathrm{d}t^2} + \omega _2^2\right) q - V(q)\\&+\,\hbox {total derivatives}. \end{aligned}$$

where *V*(*q*) is some interaction. We assume real $\omega _{1}$, $\omega _{2}$, because we are interested in ghosts (negative kinetic and potential energy), not in tachyonic instabilities (potential unstable with respect to the kinetic term). The $-$ sign means that the ghost is the state with larger $\omega$; we choose $\omega _1> \omega _2$ and do not explicitly discuss here the degenerate case $\omega _1=\omega _2$.

Ostrogradski introduced an *auxiliary* coordinate $q_2$ that allows one to describe the 4-derivative oscillator in canonical Hamiltonian form (see also ref. [21] for a review of this method):

3 $$\begin{aligned} \begin{aligned}&q_1 = q,\quad p_1 = \frac{\delta \mathscr {L}}{\delta \dot{q}_1} =(\omega _1^2+\omega _2^2)\dot{q} + \dddot{q},\\&q_2=\lambda \dot{q},\quad p_2 = \frac{\delta \mathscr {L}}{\delta \dot{q}_2} = -\frac{\ddot{q}}{\lambda }, \end{aligned} \end{aligned}$$

where for a generic variable *x* we have introduced the variational derivative

4 $$\begin{aligned} \frac{\delta \mathscr {L}}{\delta x} = \frac{\partial \mathscr {L}}{\partial x} - \frac{\mathrm{d}}{\mathrm{d}t} \frac{\partial \mathscr {L}}{\partial \dot{x}} +\frac{\mathrm{d}^2}{\mathrm{d}t^2} \frac{\partial \mathscr {L}}{\partial \ddot{x}} + \cdots . \end{aligned}$$

While Ostrogradski assumed $\lambda =1$, we introduced an arbitrary constant $\lambda$. The system in Eq. (3) can be solved for *q* and its time derivatives,

5 $$\begin{aligned} q= & {} q_1,\quad \dot{q} = \frac{q_2}{\lambda },\quad \ddot{q} = - \lambda p_2,\nonumber \\&\dddot{q} = p_1-(\omega _1^2+\omega _2^2) \frac{q_2}{\lambda }, \end{aligned}$$

and the Hamiltonian turns out to be

6 $$\begin{aligned} H= & {} \sum _{i=1}^2 p_i \dot{q}_i - \mathscr {L}= \frac{p_1 q_2}{\lambda } - \frac{\lambda ^2}{2}p_2^2 - \frac{\omega _1^2+\omega _2^2}{2\lambda ^2} q_2^2\nonumber \\&+\,\frac{\omega _1^2\omega _2^2}{2}q_1^2+V(q_1). \end{aligned}$$

In view of its first term, the classical Hamiltonian *H* has no minimal energy configuration: this is the essence of the Ostrogradski no-go classical theorem. Using the Poisson parentheses $\{~,~\}$ one computes the Hamiltonian equations of motion:

7 $$\begin{aligned} \left\{ \begin{array}{ll}\displaystyle \dot{q}_1 = \{q_1,H\} = \frac{\partial H}{\partial p_1} =\frac{q_2}{\lambda },&{}\quad \dot{p}_1 = \{p_1,H\} = -\frac{\partial H}{\partial q_1}= -\omega _1^2\omega _2^2 q_1 - V'(q_1) ,\\ \dot{q}_2 = \{q_2,H\} = \frac{\partial H}{\partial p_2}=-\lambda ^2 p_2,&{}\quad \dot{p}_2 = \{p_2,H\} = -\frac{\partial H}{\partial q_2}=-\frac{p_1}{\lambda } + \frac{\omega _1^2+\omega _2^2}{\lambda ^2} q_2. \end{array}\right. \end{aligned}$$

For any $\lambda$ they imply the classical Lagrangian equation of motion. Setting $V=0$, it is

8 $$\begin{aligned}&\left( \frac{\mathrm{d}^2}{\mathrm{d}t^2} + \omega _1^2\right) \left( \frac{\mathrm{d}^2}{\mathrm{d}t^2} + \omega _2^2\right) q\nonumber \\&\quad = \frac{\mathrm{d}^4q}{\mathrm{d}t^4}+(\omega _1^2 +\omega ^2_2)\frac{\mathrm{d}^2q}{\mathrm{d}t^2} + \omega _1^2 \omega _2^2 q=0. \end{aligned}$$

The corresponding classical solution, for given initial conditions $q_0, \dot{q}_0, \ddot{q}_0, \dddot{q}_0$ at $t=0$, is

9 $$\begin{aligned} q(t)= & {} -\frac{\omega _2^2 q_0 + \ddot{q}_0}{\omega _1^2-\omega _2^2} \cos (\omega _1 t) + \frac{\omega _1^2 q_0 + \ddot{q}_0}{\omega _1^2-\omega _2^2} \cos (\omega _2 t)\nonumber \\&-\frac{\omega _2^2 \dot{q}_0 + \dddot{q}_0}{\omega _1(\omega _1^2-\omega _2^2)} \sin (\omega _1 t) + \frac{\omega _1^2 \dot{q}_0 + \dddot{q}_0}{\omega _2(\omega _1^2-\omega _2^2)} \sin (\omega _2 t).\nonumber \\ \end{aligned}$$

This is a well-behaved oscillator without run-away issues because the positive-energy and negative-energy components are decoupled. Run-away solutions appear when they interact through a generic interaction, such as a $V\ne 0$.

### Quantizing the Ostrogradski Hamiltonian

The classical equation differs from the usual 2-derivative equation $\mathrm{d}(q+ip)/\mathrm{d}t=i\omega (q+ip)$, so that, trying to quantize the theory, we do not define the usual annihilation operator $a_i\propto q_i + i p_i$. Rather, it is convenient to define the operators $a_i$ as the coefficients of a given frequency:

10 $$\begin{aligned} q(t) = a_1 \mathrm{e}^{-i\omega _1 t} + a_2 \mathrm{e}^{-i\omega _2 t} + \hbox {h.c.} \end{aligned}$$

The $a_1,a_2$ can be expressed in terms of canonical Hamiltonian coordinates:

11 $$\begin{aligned} a_1= & {} \frac{\lambda \omega _1 \omega _2^2 q_1 - i \omega _1^2 q_2 + i p_1 \lambda - \omega _1 p_2 \lambda ^2}{2\lambda \omega _1(\omega _2^2-\omega _1^2)}, \end{aligned}$$

12 $$\begin{aligned} a_2= & {} \frac{\lambda \omega _1^2 \omega _2 q_1 - i \omega _2^2 q_2 + i p_1 \lambda - \omega _2p_2 \lambda ^2}{2\lambda \omega _2(\omega _1^2-\omega _2^2)}. \end{aligned}$$

Using the canonical quantization $[q_i,p_j]=i\delta _{ij}$ one finds the commutation relations for the $a_i$:

13 $$\begin{aligned}&[\tilde{a}_1, \tilde{a}^\dagger _1]=-1,\quad [\tilde{a}_2, \tilde{a}^\dagger _2]=1,\nonumber \\&\quad \hbox {all other commutators vanish}. \end{aligned}$$

having normalized $\tilde{a}_1 = \sqrt{2\omega _1(\omega _1^2-\omega _2^2)}a_1$ and $\tilde{a}_2 = \sqrt{2\omega _2(\omega _1^2-\omega _2^2)}a_2$. The Hamiltonian is

14 $$\begin{aligned} H=- \omega _1 \frac{ \tilde{a}_1 \tilde{a}_1^\dagger + \tilde{a}_1^\dagger \tilde{a}_1}{2} + \omega _2 \frac{\tilde{a}_2 \tilde{a}_2^\dagger +\tilde{a}_2^\dagger \tilde{a}_2}{2}. \end{aligned}$$

The state 1 with higher frequency $\omega _1>\omega _2$ is a ghost.

As better discussed later in Sect. 3.3, this system can be quantized in two different ways:

1. *Positive norm, negative energy* One redefines $\tilde{a}'_1=\tilde{a}_1^\dagger$, such that it has the usual commutation $[\tilde{a}'_1,\tilde{a}^{\prime \dagger }_1]=1$. The vacuum state $| \tilde{0} \rangle$ is defined as usual by $\tilde{a}'_1| \tilde{0} \rangle =0$ and $\tilde{a}_2| \tilde{0} \rangle =0$. By solving this condition as a differential equation for $\psi _{\tilde{0}}(q_1, q_2) = \langle q_1,q_2 | \tilde{0} \rangle$ with $p_i = - i \partial /\partial q_i$ one obtains the ground-state wavefunction:
  15 $$\begin{aligned} \psi _{ \tilde{0}}(q_1,q_2)= & {} \exp \left( -\frac{q_1^2 \omega _1 \omega _2 + q_2^2/\lambda ^2}{2} (\omega _1 - \omega _2)\right. \nonumber \\&\left. +\, i q_1 \frac{q_2}{\lambda }\omega _1 \omega _2\phantom {\frac{q_1^2 \omega _1 \omega _2 + q_2^2/\lambda ^2}{2}}\right) . \end{aligned}$$
2. *Negative norm*, *positive energy* The vacuum is now defined as $a_1| 0 \rangle =0$ and $a_2| 0 \rangle =0$. Using $p_i = - i \partial /\partial q_i$ one obtains the ground-state wavefunction
  16 $$\begin{aligned} \psi _{0}(q_1,q_2)\propto & {} \exp \left( \frac{-q_1^2 \omega _1 \omega _2 +q_2^2/\lambda ^2}{2} (\omega _1 + \omega _2)\right. \nonumber \\&\left. -\, i q_1 \frac{q_2}{\lambda }\omega _1 \omega _2\phantom {\frac{-q_1^2 \omega _1 \omega _2 +q_2^2/\lambda ^2}{2}}\right) . \end{aligned}$$

If $\lambda =1$ the situation is bad, as emphasized by [16, 17]: the positive-norm quantization gives a normalizable wavefunction $\psi _{\tilde{0}}$ but negative energies; the negative-norm quantization gives a ground-state wavefunction not normalizable in $q_2 = \dot{q}$. Excited states have the same problem.

However, as we will show in Sect. 4, consistency requires the negative-norm Dirac–Pauli representation of a canonical coordinate which roughly amounts to choosing an imaginary $\lambda$, e.g. $\lambda =- i$. One then obtains positive energy, negative norm, and a wavefunction $\psi _0(q_1,q_2)$ normalizable in $q_1$ and $q_2 =- i \dot{q}$. As we will now discuss, despite the strange *i* factor, $\dot{q}=iq_2$ as well as the Ostrogradski Hamiltonian $H = i q_2 p_1 +\cdots = \dot{q} p_1+\cdots$ is self-adjoint, so that time evolution is unitary.

## Quantum mechanics with negative norm

We here discuss quantum mechanics with negative norm from a general point of view. Negative-norm states require putting some minus sign here and there. It is convenient to be more general and consider a Hilbert-like space with generic, possibly negative, constant norm (called Krein space by mathematicians) and develop a basis-independent formalism. This will let us to clarify confusions, in particular about self-adjoint operators that are represented (in some basis) by non-hermitian matrices, allowing us to understand the unusual imaginary $\lambda$ introduced in the previous section.

We follow the notations used in general relativity, rewriting the quantum-state metric as $\langle _n | _m \rangle =\eta _{nm}$ and defining the inverse metric $(\eta )^{nm}\equiv (\eta ^{-1})_{nm}$, the contravariant ket $| ^n \rangle =\eta ^{nm}| _m \rangle$ such that $\langle ^n | ^m \rangle =\eta ^{nm}$ and $\langle ^n | _m \rangle =\delta ^n_m = \langle _n | ^m \rangle$. Summations over repeated indices are implicit. As usual, bras denote complex conjugate of kets.

A generic *state* $| \psi \rangle$ can be expanded in either the ‘covariant’ or the ‘contravariant’ basis:

17 $$\begin{aligned} \psi _n \equiv \langle _n | \psi \rangle ,\quad \psi ^n \equiv \langle ^n | \psi \rangle . \end{aligned}$$

Then

18 $$\begin{aligned} | \psi \rangle = \psi ^n | _n \rangle = \psi _n | ^n \rangle . \end{aligned}$$

A generic *linear operator* *A* can be written as a matrix in 4 different ways:

19 $$\begin{aligned}&A_{nm}\equiv \langle _n | A|_m \rangle ,\quad A^{nm}\equiv \langle ^n | A|^m \rangle ,\quad A_n{}^m\equiv \langle _n | A|^m \rangle ,\nonumber \\&\quad A^n{}_m\equiv \langle ^n | A|_m \rangle . \end{aligned}$$

Then

20 $$\begin{aligned} A ={A}^{nm}| _n \rangle \langle _m | ={A}_{nm}| ^n \rangle \langle ^m | ={A}_{n}{}^{m}| ^n \rangle \langle _m | ={A}^n{}_m| _n \rangle \langle ^m |.\nonumber \\ \end{aligned}$$

The components of the matrices are related by $A_n{}^m = \eta _{nn'} A^{n'}{}_{m'}\eta ^{m'm}$, which is an *iso-spectral* transformation: the eigenvalues do not change because the matrix *A* gets left-multiplied by $\eta$ and right-multiplied by its inverse.

The *unity operator* is represented by $1_{nm}=\eta _{nm}$ and ${1}^{nm}={\eta }^{nm}$ and expanded as

21 $$\begin{aligned} 1 = \eta ^{nm} |{}_n\rangle \langle _m | =\eta _{nm}| ^n \rangle \langle ^m | = | _n \rangle \langle ^n |=| ^n \rangle \langle _n | . \end{aligned}$$

Operator multiplication becomes, in components, $(AB)_{nm} = A_{nn'} \eta ^{n'm'} B_{m'm}$. Expectation values are given by $\langle \psi | A|\psi \rangle /\langle \psi | \psi \rangle$.

The *adjoint* $A^\dagger$ of an operator *A* is defined, as usual, as the operator such that $| \psi ' \rangle = A | \psi \rangle$ implies $\langle \psi ' | = \langle \psi | A^\dagger$. Thereby for generic matrix elements one has $\langle \psi _2 | A^\dagger |\psi _1 \rangle \equiv \langle \psi _1 | A|\psi _2 \rangle ^*$, and the relation for the components

22 $$\begin{aligned}&(A^\dagger )_{nm} = A_{mn}^*\quad \hbox {i.e.}\quad ( A^\dagger )^{nm} = A^{mn*}\nonumber \\&\quad \hbox {i.e.}\quad ( A^\dagger )_n{}^m = (A^m{}_n)^*. \end{aligned}$$

The covariant components of a self-adjoint operator *A* satisfy the usual condition: a self-adjoint operator is described by a hermitian matrix, $A_{nm}$. The same result holds for the contravariant matrix $A^{nm}$. The mixed components satisfy a different condition, where complex conjugation is supplemented by an iso-spectral transformation: $A_n{}^m = (\eta A^{*T} \eta ^{-1})_{n}{}^m$.^2^

A *self-adjoint* operator, $A^\dagger =A$ has real expectation values $\langle \psi | A|\psi \rangle /\langle \psi | \psi \rangle$, although the matrix $A^m{}_n$ that represents it can be anti-hermitian.

The mixed components directly enter into the *eigenvector equation* $A|\psi \rangle = A_\psi |\psi \rangle$:

23 $$\begin{aligned}&A_n{}^{m} \psi _m= A_{nm'}\eta ^{m'm} \psi _{m} = A_\psi \psi _n \quad \hbox {or}\nonumber \\&A^n{}_m\psi ^m= \eta ^{nn'} A_{n'm}\psi ^m = A_\psi \psi ^n \end{aligned}$$

where $A_\psi$ is the eigenvalue. Let us now consider a self-adjoint operator *H* (later it will be the Hamiltonian), with eigenstates $| E_n \rangle$ and eigenvalues $E_n$. The identity

24 $$\begin{aligned} \langle E_n | H |E_m \rangle = \langle E_n | E_m\rangle E_m = E^*_n \langle E_n | E_m\rangle \end{aligned}$$

tells that *H* can have three different kinds of eigenstates:

- $+$) orthogonal eigenstates $\langle E_n | E_m \rangle =0$ with real $E_n$ and norm $\langle E_n | E_n \rangle = +1$;
- $-$) orthogonal eigenstates $\langle E_n | E_m \rangle =0$ with real $E_n$ and norm $\langle E_n | E_n \rangle =- 1$;
- 0) pairs of complex conjugated eigenvalues, $E_n = E_m^*$ with $\langle E_n | E_m \rangle \ne 0$ and zero norm, $\langle E_n | E_n \rangle =0$.

In the classical analog, the latter possibility corresponds to a ghost which is also a tachyon, which is a different kind of instability, to be avoided even in absence of ghosts.

It is often convenient to choose a basis of eigenstates of *H*: $| _n \rangle =| E_n \rangle$. The associated contravariant states $| ^n \rangle$ then satisfy $H| ^n \rangle = E_n^*| ^n \rangle$. In this basis the space splits into two sectors: positive norm and negative norm, plus the possible pairs of zero-norm states. The two sectors experience a joint dynamics only if the initial state has a quantum entanglement among them.

### Unitary time evolution

The *evolution equation* $i \partial _t | \psi \rangle = H | \psi \rangle$ becomes

25 $$\begin{aligned} i \frac{\partial }{\partial t} \psi _n = H_{nm}\eta ^{mm'} \psi _{m'}\quad \hbox {or}\quad i \frac{\partial }{\partial t} \psi ^n = \eta ^{nn'} H_{n'm} \psi ^m. \end{aligned}$$

The norm of any state $| \psi (t) \rangle$ is conserved by time evolution if *H* is self-adjoint:

26 $$\begin{aligned} i \frac{\partial }{\partial t} \langle \psi '(t) | \psi (t) \rangle = \langle \psi ' | H-H^\dagger |\psi \rangle =0. \end{aligned}$$

A self-adjoint Hamiltonian *H* leads to unitary time evolution. The explicit solution can be written as $| \psi (t) \rangle = U(t) | \psi (0) \rangle$ with $U(t)=T \mathrm{e}^{-i\int H(t) \,\mathrm{d}t}$, where *T* is the usual time-ordering. In components,

27 $$\begin{aligned}&\psi _n(t) = U_n{}^m\psi _m(0)= U_{nm'}\eta ^{m'm} \psi _{m'}(0) \quad \hbox {or}\nonumber \\&\psi ^n (t) =U^n{}_m\psi ^m(0)= \eta ^{nn'} U_{n'm} \psi ^m(0). \end{aligned}$$

Having written generic-metric quantum mechanics in an abstract formalism that resembles as much as possible the usual positive-norm formalism, let us now emphasize the key differences. For simplicity, let us consider a time-independent *H*. One can then expand $U = \mathrm{e}^{-iHt} = \sum _{n=0}^\infty (-iHt)^n/n!$.

- Writing *U* in mixed components, $U_n{}^m$ is the naive exponentiation of the matrix $H_n{}^m$. However, the mixed components of a self-adjoint *H* do not form a hermitian matrix. Rather, the self-adjoint condition in Eq. (22) dictates that they are hermitian up to an iso-spectral transformation.
- The covariant components of a self-adjoint *H* satisfy the usual Hermiticity $H^*_{nm}=H_{mn}$. However, the covariant components $U_{nm}$ are not given by the naive matrix exponentiation of $H_{nm}$. Rather, extra metric factors appear to covariantize the expansion:
  28 $$\begin{aligned} U_{nm}= & {} \eta _{nm} + \eta _{nn'} (-i Ht)^{n'm'} \eta _{m'm}\nonumber \\&+\,\frac{1}{2} \eta _{nn'} (-i Ht)^{n'r'} \eta _{r's'} (-i Ht)^{s'm'} \eta _{m'm} + \cdots \nonumber \\ \end{aligned}$$
  Correspondingly, the unitarity condition $U^\dagger U =1$ written in covariant components is $U_{n'n}^*\eta ^{n'm'} U_{m'm} = \eta _{nm}$, while in mixed components one gets the usual $U^{k*}_{~\,n} \, U_k{}^m=\delta _m^n$.

Practical computations often employ perturbation theory, which can now easily be generalized to generic norm. Decomposing $H=H_0+V(t)$, the interaction picture is related to the Schrödinger picture as $A_I = \mathrm{e}^{iH_0 t} A \mathrm{e}^{-iH_0 t}$ where *A* is any operator (including *V*). Time evolution is given by

29 $$\begin{aligned} U_I (t_i,t_f)= & {} \mathrm{T}\, \mathrm{e}^{-i\int _{t_i}^{t_f} \mathrm{d}t \,V_I(t)} =1-i\int _{t_i}^{t_f} \mathrm{d}t'\,V_I(t')\nonumber \\&-\, \int _{t_i}^{t_f} \mathrm{d}t'\int _{t_i}^{t'}\mathrm{d}t'' \,V_I(t') V_I(t'')+\cdots . \end{aligned}$$

The above explicit form of $U_I$ shows that the energy conserved by quantum evolution (up to the usual quantum uncertainty $\Delta t \,\Delta E \ge \hbar$) are the eigenvalues of *H*. Let us consider for example a time-independent interaction *V* and an initial state and a final state which are energy eigenstates with eigenvalues $E_i$ and $E_f$. Defining $V^f{}_i=\langle ^f | V |{}_i\rangle$, at first order one has

30 $$\begin{aligned} |\langle ^f | U|_i \rangle |^2&\simeq \left| \int _0^t \mathrm{d}t' ~\mathrm{e}^{i(E_f-E_i)t' } V^f{}_i \right| ^2 \nonumber \\&= \frac{4| V^f{}_i|^2}{|E_f-E_i|^2}\sin ^2\frac{(E_f-E_i)t}{2}\nonumber \\&\mathop {\simeq }\limits ^{t\rightarrow \infty } 2\pi t | V^f{}_i|^2 \delta (E_f-E_i). \end{aligned}$$

This means that energy conservation reads $E_f=E_i$, up to the usual quantum uncertainty $1/(t_f-t_i)$. Higher order corrections give the usual sum over intermediate quasi-on-shell states.

### Example: the indefinite-norm two-state system

Let us consider a two-state system: $| _+ \rangle$ with positive unit norm, and $| _- \rangle$ with negative unit norm. Without loss of generality, by redefining the relative phase of the two states and adding a constant overall energy, one can trivially write the most generic self-adjoint Hamiltonian as31having used $| ^\pm \rangle =\pm | _\pm \rangle$. We see that the $H_{nm}$ components are hermitian, unlike the $H_n{}^m$ components. The eigenvalues of *H* are $E_\pm = \pm E$ with $E=\sqrt{E_R^2-E_I^2}/2$. The corresponding eigenstates are

32 $$\begin{aligned}&| _{E_+} \rangle = \sqrt{\frac{\gamma + 1}{2}} | _+ \rangle -i \sqrt{\frac{\gamma - 1}{2}} | _- \rangle , \nonumber \\&| _{E_-} \rangle =i\sqrt{\frac{\gamma - 1}{2}} | _+ \rangle + \sqrt{\frac{\gamma + 1}{2}} | _- \rangle , \end{aligned}$$

where $\gamma = 1/\sqrt{1-E_I^2/E_R^2}$ is a ‘boost factor’ that substitutes the usual mixing angle.

- If $E_I< E_R$ the eigenvalues of *H* are real, the orthogonal eigenvectors satisfy $\langle _{E_\pm } | _{E_\pm } \rangle = \pm 1$, and tend to get closer to the ‘light-cone’ of zero-norm states as $E_I$ increases. The components of $U= \mathrm{e}^{-i H t}$ oscillate in time: 33 The unusual feature is that $|\langle _\pm | U|_\pm \rangle |^2$ oscillates between 1 and $\gamma ^2\ge 1$.
- In the critical case, $E_R=E_I$, such that $\gamma =\infty$, the two eigenstates become degenerate with energy $E=0$. The two eigenvectors also become degenerate, and parallel to the zero-norm state $\propto | _+ \rangle +i | _- \rangle$. The evolution operator is 34 This exemplifies a more general result: zero-norm eigenstates with complex eigenvalues appear when, increasing the interaction, a level crossing between a positive-norm and a negative-norm eigenstate takes place; the Hamiltonian becomes degenerate at the critical transition.
- If the interaction $E_I$ is strong enough, $E_I >E_R$, one has a pair of complex conjugated eigenvalues, with zero-norm eigenvectors that satisfy $\langle _{E_+} | _{E_-} \rangle =1$ and describe tachyonic ghosts: their time-evolution factor $\mathrm{e}^{-iE_\pm t}$ also contains a real exponential, in analogy to tachyonic states present in positive-norm theories. In the extreme limit $E_I\gg E_R$ the eigenvalues of *H* are $\pm i E_I/2$, and the time evolution operator is 35

This runaway happens whenever *H* has a pair of complex eigenvalues $E_+=E_-^*$, as clear writing time evolution in terms of energy eigenstates, $|\psi (t)\rangle = \psi ^{E_+} \mathrm{e}^{-i E_+ t} |{}_{E_+}\rangle + \psi ^{E_-} \mathrm{e}^{-i E_- t} |{}_{E_-}\rangle$. Both the norm of $| \psi (t) \rangle$ and the real expectation value of *H* are preserved by time evolution:

36 $$\begin{aligned} \frac{ \langle \psi (t) | H | \psi (t) \rangle }{\langle \psi (t) | \psi (t) \rangle } = \frac{E_+ \psi ^{E_+} \psi ^{E_-*} +\hbox {c.c.} }{\psi ^{E_+} \psi ^{E_-*} + \hbox {c.c.}}. \end{aligned}$$

### The negative-norm harmonic oscillator

We here study the concrete system that lies at the basis of perturbative Quantum Field Theory: the harmonic oscillator. As discussed by Lee and Wick [22, 23] it admits two inequivalent quantizations: positive norm, and indefinite norm.

Let us first recall the standard oscillator, described (up to irrelevant constants) by the Hamiltonian $H = \frac{1}{2} (p^2+q^2)$ with $[q,p]=i$. Defining

37 $$\begin{aligned} a=\frac{q+ip}{\sqrt{2}},\quad a^\dagger =\frac{q-ip}{\sqrt{2}} \end{aligned}$$

one has $[a,a^\dagger ]=1$ and $H=(a a^\dagger +a^\dagger a) /2$.

Let us next consider a more general system described by the following Hamiltonian and commutation relations:

38 $$\begin{aligned} H = s_H \frac{a^\dagger a+aa^\dagger }{2},\quad [a,a^\dagger ]=s. \end{aligned}$$

For $s=s_H=1$ this reduces to the usual oscillator. We now show that $s=s_H=-1$ defines another consistent positive-energy theory. The symbol $a^\dagger$ here indicates the adjoint of *a*, which generalizes the Hermitian conjugate to negative norm.

We again define the vacuum as $a| 0 \rangle =0$ and the excited states as $| _n \rangle =a^\dagger | _{n-1} \rangle /\sqrt{n}= (a^\dagger )^n| 0 \rangle /\sqrt{n!}$. Its inverse is $a| _n \rangle = s\sqrt{n}| _{n-1} \rangle$. The state metric is $\eta _{nm}\equiv \langle _m | _n \rangle =s^{n}\delta _{nm}$. The norm is determined by the dynamics, and odd states have negative norm for $s=-1$. The inverse metric is $\eta ^{nm}=s^{-n}\delta _{nm}$ and the contravariant states are $| ^n \rangle = s^{-n} | _n \rangle$. In components one has39
40In components the commutation relations read

41 $$\begin{aligned}{}[a,a^\dagger ]_{nm}= & {} (a\cdot \eta \cdot a^\dagger - a^\dagger \cdot \eta \cdot a)_{nm}= s^{n+1} \delta _{nm}\nonumber \\= & {} s\eta _{nm}\quad \hbox {i.e.}\quad [a,a^\dagger ] = s \sum _n | _n \rangle \langle ^n | = s 1 \end{aligned}$$

and the Hamiltonian is

42 $$\begin{aligned}&H_{nm} = \left( n+\frac{1}{2}\right) s_H s^{n+1} \delta _{nm}= E_n \eta _{nm}\quad \nonumber \\&\quad \hbox {i.e.}\quad H = \sum _{n=0}^\infty E_n | _n \rangle \langle ^n | \end{aligned}$$

where $E_n = (n+\frac{1}{2}) s s_H$ are the Hamiltonian eigenvalues, $H| _n \rangle =E_n | _n \rangle$. We see that positive-energy eigenvalues are obtained for $s=s_H=1$ (the usual case with positive *H* and positive norm), but also for $s=s_H=-1$ (negative *H* and negative norm).

Concerning the negative-norm case, $s=-1$, notice that the harmonic oscillator does not predict tachyonic ghosts with zero norm. Furthermore the matrix elements $a_{n}{}^m$ are not the hermitian conjugates of $(a^\dagger )_n{}^m$, such that the operators $q=(a+a^\dagger )/\sqrt{2}$ and $p=i(a^\dagger -a)/\sqrt{2}$ are represented by matrices $q_n{}^m$ and $p_n{}^m$ which are not Hermitian. This is why various authors who look at these matrices improperly speak of ‘anti-Hermitian’ operators. Nevertheless, *q* and *p* are self-adjoint operators. We will now find their coordinate representation.

### The negative-norm coordinate representation

Starting from the harmonic oscillator, we now describe a more general representation of a pair of canonical coordinate variables *q*, *p*. Parity flips $q\rightarrow -q$ and $p\rightarrow - p$. In the harmonic oscillator case, this means $a\rightarrow -a$ and $a^\dagger \rightarrow -a^\dagger$: so eigenstates $| _n \rangle$ with even (odd) *n* are even (odd) under parity. In the negative-norm quantization, states with odd *n* also have negative norm. Going to the coordinate wavefunction representation (we use the notation *x* for the coordinate, which later will become field space), this means that the norm is

43 $$\begin{aligned} \langle \psi ' | \psi \rangle= & {} \int \mathrm{d}x\, [ \psi ^{\prime *}_\mathrm{even}(x) \psi _\mathrm{even}(x) -\psi ^{\prime *}_\mathrm{odd}(x)\psi _\mathrm{odd}(x)] \nonumber \\= & {} \int \mathrm{d}x \, \psi ^{\prime *}(x) \psi (-x). \end{aligned}$$

The corresponding unit operator is $1 = \int \mathrm{d}x | -x \rangle \langle x |$. Switching to the formalism appropriate for generic norm, one has the norm $\langle _{x'} | _x \rangle = \delta (x+x')$. Thereby the contravariant state is $| ^x \rangle =| _{-x} \rangle$ and it satisfies the usual $\langle ^{x'} | _x \rangle =\delta (x-x')$. As already discussed in the text surrounding Eq. (17), a state can be expanded as $| \psi \rangle = \int \mathrm{d}x \, \psi (_x)| ^x \rangle =\int \mathrm{d}x\,\psi (^x)| _x \rangle$ with $\psi (_x)=\langle _x | \psi \rangle$ and $\psi (^{x}) =\langle ^x | \psi \rangle =\psi (_{-x})$.

What is emerging from the harmonic oscillator computation is a more general structure: a coordinate space representation of a pair *q*, *p* of conjugated canonical variables that differs from the usual positive-norm representation

44 $$\begin{aligned} { q | x \rangle = x | x \rangle ,\quad p | x \rangle = +i \frac{\mathrm{d}}{\mathrm{d}x} | x \rangle }, \end{aligned}$$

which implies $\langle x | p|\psi \rangle = (- i \mathrm{d}/\mathrm{d}x)\psi (x)$ so that it satisfies $\langle x | [q,p]|\psi \rangle = i \langle x | \psi \rangle$.

The negative-norm coordinate representation, originally discussed by Dirac [18] and Pauli [19], is45Although *q* looks anti-hermitian, taking into account the extra *i* as well as the negative norm, these unusual features combine to form a self-adjoint *q*:

46 $$\begin{aligned} \langle _{x'} | q^\dagger |_x \rangle= & {} \langle _x | q|_{x'} \rangle ^* = [ix' \delta (x+x') ]^*\nonumber \\= & {} ix \delta (x+x') = \langle _{x'} | q|_x \rangle . \end{aligned}$$

This means that $\langle \psi | q|\psi \rangle =\int \mathrm{d}q~\psi ^*(-q) iq\,\psi (q)$ is real. A similar result holds for *p*. When acting on wavefunctions one has $\langle _x | q|\psi \rangle =-ix \psi (_x)$ and $\langle _x | p|\psi \rangle = (+\mathrm{d}/\mathrm{d}x) \psi (_x)$, giving the desired $[q,p]=i$ commutator. Defining momentum eigenstates as $p| _p \rangle =ip| _p \rangle$ one finds $\langle _q | _p \rangle = \mathrm{e}^{ipq}/\sqrt{2\pi }$, $\langle _{p'} | _p \rangle = \delta (p+p')$. The operator *q* acts as $\langle _q | q|_p \rangle = (-\mathrm{d}/\mathrm{d}p) \langle _q | _p \rangle$. One can again define $| ^p \rangle = | _{-p} \rangle$ such that $1=\int \mathrm{d}p \, | ^p \rangle \langle _p |$.

The *i* factor that differentiates the usual representation from the Dirac–Pauli representation has an impact on the time-inversion parity. As usual, a positive-energy spectrum requires that the time inversion symmetry is anti-unitary. Then, in the Dirac–Pauli quantization *q* is naturally *T*-odd and *p* is naturally *T*-even (while the opposite holds in the usual quantization, unless *T* is defined adding ad hoc extra signs). This will play a key role in Sect. 4.

We are now ready to come back to the harmonic oscillator. Inserting into the condition $\langle x | a|0 \rangle =0$ the standard positive-norm representation such that $a = (q+ip)/\sqrt{2} =(x +s\, \mathrm{d}/\mathrm{d}x)/\sqrt{2}$ gives a differential equation which implies the ground-state wavefunction $\psi _0(x)\propto \mathrm{e}^{-sx^2/2}$. This is normalizable for $s=1$ (positive norm) and non-normalizable for $s=-1$, where *s* was defined in Eq. (38). This problem was emphasized e.g. by Woodard [16, 17] who thereby dismissed the negative-norm quantization as purely formal. However, the problem arises because the positive-norm representation of *q*, *p* was used together with the negative-norm oscillator: the problem is just a manifestation of the inconsistency of the assumptions. Consistency requires that the negative-norm harmonic oscillator must be accompanied by the negative-norm Dirac–Pauli coordinate space representation of the self-adjoint *q*, *p* operators, Eq. (45). Then the condition $\langle x | a|0 \rangle =0$ leads to a normalizable wavefunction for the ground state $\psi _0 \propto \mathrm{e}^{-x^2/2}$, as well as for the excited states. The Dirac–Pauli choice thereby provides a self-consistent description of the negative-norm oscillator. Furthermore, as discussed in the next section, in the 4-derivative case the Dirac–Pauli representation is required by simple considerations.

**Table 1:** Coordinate representations of a pair of canonical variables $[q,p]=i$, and the associated ground-state wavefunctions for the positive-energy harmonic oscillator

Norm	$\langle x | q|\psi \rangle$	$T\hbox {-parity}$	$\langle x | p|\psi \rangle$	$T\hbox {-parity}$	Harmonic oscillator with $E>0$
Positive	$x\psi (x)$	Even	$-i\,d\psi /d x$	Odd	$\psi _0(q) \propto \mathrm{e}^{-q^2/2}$ and $H = +\frac{1}{2} (q^2+p^2)$
Indefinite	$-ix\psi (x)$	Odd	$d\psi /d x$	Even	$\psi _0(q) \propto \mathrm{e}^{-q^2/2}$ and $H =-\frac{1}{2} (q^2+p^2)$

## For four derivatives Dirac–Pauli is desirable

As discussed in the previous section, and as summarized in Table 1, quantum mechanics has two faces: a canonical coordinate can be represented

(i) in the usual way with positive norm;
(ii) in the Dirac–Pauli way, with negative norm, Eq. (45).

As we now show, for theories with four derivatives the latter quantization choice (which, in the gravitational case, corresponds to a renormalizable theory with positive energy) is desirable.

A single 4-derivative real coordinate *q*(*t*) contains two degrees of freedom. In the Ostrogradski procedure (Sect. 2) one rewrites the theory as a Hamiltonian system of two canonical coordinates, $q_1=q$ and $q_2=\lambda \dot{q}$. The key new feature that arises in 4-derivative theories is that $\dot{q}$ becomes an extra canonical coordinate. In the classical theory $q_2$ is just an auxiliary variable, and $\lambda$ is an irrelevant constant: Ostrogradski used $\lambda =1$.

In the quantum theory, $q_1$ and $q_2$ are operators that allow one to define the basis $| q_1, q_2 \rangle$. We now show that the usual quantization must be used for $q_1$ and that the Dirac–Pauli quantization must be chosen for $q_2$, which is equivalent to (and more transparent than) fixing an imaginary $\lambda$ and using the canonical representation.

As usual, the operator $q_1=q$ is invariant under time-reversal $t\rightarrow - t$, and thereby it can follow the usual *T*-even representation. On the other hand, the operator $\dot{q}$ is *T*-odd, because of the time derivative: the time-inversion operator *T* transforms it as $T \dot{q} T^{-1}=-\dot{q}$. This is the novel key feature.

Taking into account that *T* is anti-unitary, one can equivalently define a usual *T*-even coordinate $q_2 = \lambda \dot{q}_1$ by choosing an imaginary $\lambda$.^3^ However, it is simpler to forget the $\lambda$ factors and just declare that the self-adjoint operator $\dot{q}$ is *T*-odd and thereby it follows the *T*-odd Dirac–Pauli representation. Then the Ostrogradski Hamiltonian of Eq. (6) is *T*-even. The states satisfy $T| q,\dot{q} \rangle = | q,\dot{q} \rangle$ since $\dot{q}$ has imaginary eigenvalues and since *T* is anti-unitary.

The strange extra factor of *i* has been justified from first principles. With hindsight, it was not so strange. After all, it is well known that the self-adjoint spatial gradient is $i\vec \nabla$ rather than $\vec \nabla$. In a relativistic theory, one could have guessed that similarly the self-adjoint time derivative is $i\partial /\partial t$ rather than $\partial /\partial t$. Loosely speaking, while from a classical perspective $\dot{q}$ was the natural auxiliary variable, from a quantum perspective the natural extra coordinate operator is $i\dot{q}$.^4^

Using the Heisenberg representation, one has $q(t) = U^\dagger (t) q(0) U(t)$ and $\dot{q} = -i[q,H] = U^\dagger (t) \dot{q}(0) U(t)$ with unitary *U*, so *q*(*t*) keeps real eigenvalues and $\dot{q}(t)$ keeps imaginary eigenvalues at any *t* (these statements are not contradictory, given that *q*(*t*) also depends on $p_1(0)$ and $p_2(0)$).

### The frequency eigenstates

We conclude this section by computing what the Dirac–Pauli representation adopted for $q_2=\dot{q}$ implies for the frequency eigenstates. We restart from the Hamiltonian Eq. (6) and bring it in diagonal form,

52 $$\begin{aligned} H = -\frac{1}{2} \left( \tilde{p}^{ 2}_1 \tilde{\lambda }^{ 2} +\omega _1^2 \frac{\tilde{q}_1^{ 2}}{\tilde{\lambda }^{ 2}}\right) + \frac{1}{2} (\tilde{p}^{2}_2 + \omega _2^2 \tilde{q}^{ 2}_2) \end{aligned}$$

through the canonical transformation

53 $$\begin{aligned}&q_1 =\frac{ \tilde{q}_2 - \tilde{\lambda } \tilde{p}_1/\omega _1}{\sqrt{\omega _1^2 - \omega _2^2}},\quad {q_2\over \lambda } = \frac{\tilde{p}_2 - \omega _1\tilde{q}_1/\tilde{\lambda }}{\sqrt{\omega _1^2 - \omega _2^2}},\nonumber \\&\quad p_1 = \omega _1\frac{\omega _1 \tilde{p}_2 -\omega _2^2\tilde{q}_1/\tilde{\lambda }}{\sqrt{\omega _1^2 - \omega _2^2}},\quad p_2 \lambda = \frac{\omega _2^2 \tilde{q}_2-\omega _1 \tilde{\lambda } \tilde{p}_1 }{\sqrt{\omega _1^2 - \omega _2^2}}, \end{aligned}$$

which satisfies $q_1 p_1 - q_2 p_2 = \tilde{p}_2 \tilde{q}_2-\tilde{p}_1\tilde{q}_1$. For the sake of generality, we here allow for generic factors $\lambda$ and $\tilde{\lambda }$. The non-vanishing commutators, $[\tilde{q}_1,\tilde{p}_1]=i$ and $[\tilde{q}_2,\tilde{p}_2]=i$, can be rewritten in terms of $\tilde{a}_{2} =\sqrt{\omega _2/2} ( \tilde{q}_{2}+ i \tilde{p}_{2}/\omega _2)$ and of $\tilde{a}_1 =\sqrt{\omega _1/2}(\tilde{q}_1/\tilde{\lambda }- i \tilde{\lambda }\tilde{p}_1/\omega _1)$ reproducing the Hamiltonian of Eq. (14) and the commutators of Eq. (13). The ground-state wave function is easily computed imposing $\langle \tilde{q}_1,\tilde{q}_2 | \tilde{a}_{1,2}|0 \rangle =0$ finding

54 $$\begin{aligned} \psi _0(\tilde{q}_1,\tilde{q}_2) \propto \exp \left[ -\omega _2 \frac{\tilde{q}^2_2}{2}+\omega _1 \frac{\tilde{q}^2_1}{2\tilde{\lambda }^{2}}\right] . \end{aligned}$$

For $\tilde{\lambda }=1$ it is not normalizable [43, 44]. It is normalizable if instead $|\mathrm{Im}\,\tilde{\lambda }|>|\mathrm{Re}\,\tilde{\lambda }|$.

The Dirac–Pauli representation for $q_2,p_2$ corresponds to imaginary $\lambda$. Imposing that $q_2, p_1$ are *T*-odd and that $q_1,p_2$ are *T*-even (i.e. that $q_2$ and $p_2$ have the unusual *T* parity) implies that $\tilde{q}_1, \tilde{p}_2$ are *T*-odd and that $\tilde{q}_2,\tilde{p}_1$ are *T*-even (i.e. that the canonical coordinates of the negative-norm mode $\tilde{q}_1$ and $\tilde{p}_1$ have the unusual *T* parity). This is obtained for imaginary $\tilde{\lambda }$.

As a check, let us connect the $q_1,q_2$ basis with the $\tilde{q}_1,\tilde{q}_2$ basis for generic $\lambda$ and $\tilde{\lambda }$. It is convenient to start from the *T*-odd basis $\tilde{q}_1,\tilde{p}_2$, in which the ground-state wavefunction is

55 $$\begin{aligned} \psi _0(\tilde{q}_1,\tilde{p}_2) \propto \exp \left[ - \frac{\tilde{p}^2_2}{2\omega _2}+\omega _1 \frac{\tilde{q}^2_1}{2\tilde{\lambda }^{2}}\right] . \end{aligned}$$

Next, the transition to the *T*-odd variables $p_1, q_2$ is simply

56 $$\begin{aligned} \langle p_1,q_2 | \tilde{q}_1,\tilde{p}_2 \rangle\propto & {} \delta \left( \frac{\tilde{q}_1}{\tilde{\lambda }} -\frac{p_1 - q_2\omega _1^2/\lambda }{\omega _1\sqrt{\omega _1^2-\omega _2^2}}\right) \nonumber \\&\times \,\delta \left( \tilde{p}_2 -\frac{p_1 - q_2\omega _2^2/\lambda }{\sqrt{\omega _1^2-\omega _2^2}}\right) . \end{aligned}$$

Inserting the change of variables dictated by the $\delta$ functions into $\psi _0(\tilde{q}_1,\tilde{p}_2)$ one obtains

57 $$\begin{aligned}&\psi _0(p_1,q_2) \nonumber \\&\quad \propto \exp \left[ -\frac{p_1^2 + 2\omega _1 \omega _2 p_1 q_2/\lambda -\omega _1 \omega _2 (\omega _1^2+\omega _1\omega _2 +\omega _2^2)(q_2/\lambda )^2}{2\omega _1\omega _2(\omega _1+\omega _2)}\right] \nonumber \\ \end{aligned}$$

where $q_2$ and $p_1$ are both complex and linked by $\mathrm{Re}\,p_1 = \omega _1^2 \mathrm{Re}\,(q_2/\lambda )$ and $\mathrm{Im}\,p_1 = -\omega _2^2 \mathrm{Im}\,(q_2/\lambda )$. $\psi _0$ can be trivially analytically continued to real $p_1,q_2$. For $\lambda =\pm i$ it remains a bounded Gaussian. Finally, one performs the Fourier transform from $p_1$ to $q_1$, obtaining from $\psi _0(p_1,q_2)$ the ground-state wavefunction $\psi _0(q_1,q_2)$, which agrees with Eq. (16). The same equality holds for excited states, which can be computed acting with creation operators on the ground state.

In the limit $\omega _1=\omega _2$ one gets the critical situation described in Sect. 3.2.

## Path-integral quantization

We now present the path-integral quantization of the same 4-derivative theory.

### Path integral for generic norm

Our generic-norm formalism makes easy to write down the equivalent path integral formalism, an issue already considered in [27]. Inserting $1 = \int \mathrm{d}q \, |^q\rangle \langle _q|$ at intermediate times $t_m = t_i +m\, \mathrm{d}t$ one has

58 $$\begin{aligned} \langle ^{q_f, t_f}|_{q_i, t_i} \rangle = \prod _m \int \mathrm{d}q_m \langle ^{q_{m+1}, t_{m+1} }| _{q_m, t_m}\rangle . \end{aligned}$$

Each step $\langle ^{q_{m+1}, t_{m+1} }| _{q_m, t_m}\rangle$ can be evaluated as

59 $$\begin{aligned} \langle ^{q_{m+1}}| \mathrm{e}^{-i H \mathrm{d}t} | _{q_m} \rangle= & {} \int \mathrm{d}p_m \,\langle ^{q_{m+1}}| ^{p_m}\rangle \langle _{p_m} |\mathrm{e}^{-i H \mathrm{d}t}|{}_{q_m}\rangle \nonumber \\= & {} \int \frac{\mathrm{d}p_m}{2\pi } \mathrm{e}^{i [p_m (q_{m+1}-q_m)-H_\mathrm{cl} \mathrm{d}t]}, \end{aligned}$$

having defined

60 $$\begin{aligned} H_\mathrm{cl} \equiv \frac{\langle _p | H|_q \rangle }{\langle _p | _q \rangle } \end{aligned}$$

and using $\langle ^q | ^p \rangle = \mathrm{e}^{ipq}/\sqrt{2\pi }$ and $\langle _p | _q \rangle =\mathrm{e}^{-ipq}/\sqrt{2\pi }$. The final result is the path integral

61 $$\begin{aligned}&\langle ^{q_f, t_f} | _{q_i, t_i} \rangle = \int {Dq\,Dp}~\mathrm{e}^{i \int \mathrm{d}t [p\dot{q} - H_\mathrm{cl}]} \nonumber \\&\quad \hbox {where}\ Dq\,Dp =\lim _{\mathrm{d}t\rightarrow 0} \prod _m \frac{\mathrm{d}q_m \mathrm{d}p_m}{2\pi } \end{aligned}$$

and with boundary conditions $q(t_i)=q_i$, $q(t_f)=q_f$.

### Path integral for 4-derivative quantum theories

Applying the generic path integral of Eq. (61) to the 4-derivative oscillator in the canonical Ostrogradski formalism, one gets the transition amplitude

62 $$\begin{aligned}&\langle ^{q_{1f},q_{2f},t_f} | _{q_{1i},q_{2i},t_i} \rangle \propto \int Dq_1 Dp_1 Dq_2 Dp_2 \, \nonumber \\&\quad \times \exp \left[ {i \int \mathrm{d}t \left[ p_1\dot{q}_1 + p_2\dot{q}_2 - H_\mathrm{cl}+ J_1 q_1 + J_2 q_2\right] }\right] \nonumber \\ \end{aligned}$$

where for generality we added currents $J_{1,2}$ (such that acting with functional derivatives with respect to them one can form more general matrix elements of time-ordered operators; $J_1$ is *T*-even and $J_2$ is *T*-odd). The Dirac–Pauli representation for $\dot{q}$ manifests in two ways:

1. A propagator with an unusual $-$ in its external state.

Rewriting the transition amplitude in the usual positive-norm formalism, it acquires an usual $-$ sign, becoming $\langle {q_f,-\dot{q}_f,t_f} | {q_i,\dot{q}_i,t_i} \rangle$. In the limit $t_f\rightarrow t_i$ one has $\langle ^{q_f,\dot{q}_f} | _{q_i,\dot{q}_i} \rangle = \delta (q_f-q_i) \delta (\dot{q}_f-\dot{q}_i)$, so that the unusual $-$ sign is equivalent to the Dirac–Pauli negative norm.^5^ Furthermore, the *T*-odd nature of $\dot{q}$ is hardwired in the path integral, as a geometrical feature. For each trajectory *q*(*t*) with boundary conditions $q(t_{i,f}) = q_{i,f}$ and $\dot{q}(t_{i,f}) =\dot{q}_{i,f}$ the time-inverted trajectory has the same action and the following boundary conditions:

64 $$\begin{aligned} q_i \rightarrow q_f,\quad q_f\rightarrow q_i,\quad \dot{q}_i\rightarrow - \dot{q}_f,\quad \dot{q}_f\rightarrow - \dot{q}_i. \end{aligned}$$

Thereby the propagator given by the path integral satisfies the identity

65 $$\begin{aligned} \langle q_f, -\dot{q}_f,t_f | q_i, \dot{q}_i,t_i \rangle =\langle q_i, \dot{q}_i,t_f | q_f,- \dot{q}_f,t_i \rangle \end{aligned}$$

which is equivalent to the operator identity $\langle \psi _f | \psi _i \rangle =\langle T\psi _i | T\psi _f \rangle$ given that $T| q,\dot{q},t \rangle =| q,\dot{q},-t \rangle$.

2. An unusual classical Hamiltonian.

Inserting the Ostrogradski Hamiltonian of Eq. (6) in the generic path integral of Eq. (61) one gets the following classical Hamiltonian:

66 $$\begin{aligned} H_\mathrm{cl}= & {} \frac{ \langle _{p_1,p_2} | H|_{q_1,q_2} \rangle }{\langle _{p_1,p_2} | _{q_1,q_2} \rangle }\nonumber \\= & {} i p_1 q_2 + \frac{p_2^2}{2}+\frac{\omega _1^2+\omega _2^2}{2} q_2^2 + \frac{\omega _1^2\omega _2^2}{2} q_1^2+V(q_1).\nonumber \\ \end{aligned}$$

This is the same as Eq. (6) with $\lambda =-i$. $H_\mathrm{cl}$ can be complex because $q_2,p_2$, in the Dirac–Pauli representation, have complex eigenvalues.^6^ Thanks to the unusual *i*, it is invariant under time-reversal.

Let us now try to evaluate the path integral. As usual, one can perform the Gaussian $Dp_1 Dp_2$ path integrals. The $Dp_1$ path integral formally gives the Dirac delta function $\delta (q_2-\lambda \dot{q}_1)$, allowing to eliminate the $Dq_2$ path integral, leaving

67 $$\begin{aligned}&\langle ^{q_{f},\dot{q}_{f},t_f} | _{q_{i},\dot{q}_{i},t_i} \rangle \nonumber \\&\quad \propto \int Dq \, \exp \left[ {i \int \mathrm{d}t \left[ \mathscr {L}(q) + J_1 q +J_2 \lambda \dot{q}\right] }\right] , \end{aligned}$$

where $\mathscr {L}$ coincides with the original 4-derivative Lagrangian. By partial integration, the source term for $\dot{q}$ can be transformed into a source for *q* or for $\ddot{q}$ (like in the auxiliary-field method). This computation however has three problems:

1. The $Dp_1$ path integral is, in general, divergent. Thereby the subsequent result is only formal.
2. The $\delta (q_2-\lambda \dot{q}_1)$ always vanishes if $q_1$ and $q_2$ are real. Thereby the $Dq_2$ path integral is only formal.
3. Once interactions are turned on, the Lagrangian admits classical runaway solutions, reflected in the path integral.

Given that the theory is well defined in the operator formalism, somehow this path integral must have a sense.

### Euclidean path integral for 4-derivative quantum theories

A sensible path integral is found by starting from Eq. (62) and continuing it to Euclidean time, $it = t_E$, such that $\mathrm{d}q/\mathrm{d}t = i \, \mathrm{d}q/\mathrm{d}t_E$ i.e. $\dot{q} = i q'$. One gets the Euclidean path integral

68 $$\begin{aligned}&\langle ^{q_{1f},q_{2f},t_{Ef}} | _{q_{1i}, q_{2i},t_{Ei}} \rangle \propto \int Dq_1Dq_2Dp_1 Dp_2\nonumber \\&\quad \times \exp \left[ \int \mathrm{d}t_E (i p_1 q'_1 + i p_2 q'_2-H_\mathrm{cl} + J_1 q_1+J_2 q_2)\right] .\nonumber \\ \end{aligned}$$

Now the $Dp_1$ integral is convergent and gives $\delta (q_2-q'_1)$, such that the $Dq_2$ path integral just fixes $q_2 = q'_1$. Next, the remaining terms in $H_\mathrm{cl}$ are a sum of positive squares so all other integrals are convergent. Performing them one finds the Lagrangian Euclidean path integral:69where the classical Euclidean Lagrangian corresponding to Eq. (3) is

70 $$\begin{aligned} \mathscr {L}_E =\frac{1}{2} \left( \frac{\mathrm{d}^2q}{\mathrm{d}t_E^2}\right) ^2 + \frac{\omega _1^2+\omega _2^2}{2}\left( \frac{\mathrm{d}q}{\mathrm{d}t_E}\right) ^2+ \frac{\omega _1^2 \omega _2^2}{2}q^2+V(q).\nonumber \\ \end{aligned}$$

Let us now check the result. The classical free solution is

71 $$\begin{aligned} q(t_E) = a_1 \mathrm{e}^{-\omega _1 t_E}+a_2 \mathrm{e}^{-\omega _2 t_E}+ b_1\mathrm{e}^{\omega _1 t_E}+b_2 \mathrm{e}^{\omega _2 t_E}. \end{aligned}$$

It already contains runaway exponentials, characteristic of any Euclidean theory. Interactions compatible with the positivity of the action lead to an equally good path integral. By imposing the boundary conditions $q= q' =0$ at $t_{Ei}=-\infty$ and evaluating the classical action, one finds the normalizable ground-state wave function

72 $$\begin{aligned}&\langle q, q', t_E=0 | 0,0,t_E=-\infty \rangle \nonumber \\&\quad \propto \exp \left[ -\frac{q^2 \omega _1 \omega _2 + q^{\prime 2}}{2} (\omega _1 + \omega _2) + q q'\omega _1 \omega _2\right] . \end{aligned}$$

This agrees with the ground-state wavefunction $\psi _0(q_1,q_2)$ in Eq. (16), which was computed in the Dirac–Pauli formalism in Minkowski space, after identifying $q=q_1$ and $q'=q_2$. In other words, $q' = \mathrm{d}q/\mathrm{d}t_E = -i \mathrm{d}q/\mathrm{d}t$ coincides with $q_2$, as computed for $\lambda =- i$. The novel feature introduced by four derivatives is that $q'$ must *not* be continued into an imaginary $-i \dot{q}$ (which would give divergent wavefunctions), because it already describes the *T*-odd variable $q_2$, which contains the Dirac–Pauli *i* factor of Eq. (45).^7^ The final result is that the Minkowskian theory is an unusual analytic continuation of the Euclidean theory.

## Interactions, quantum field theory, probability

Summarizing, we so far considered a 4-derivative harmonic oscillator. One might think that we achieved nothing [43, 44]. After all, a classical 4-derivative harmonic oscillator has no runaway problems, see Eq. (9), given that it splits into two decoupled oscillators, one with negative energy and one with positive energy. The classical trouble starts when they interact. In this section we will explain that we have achieved instead something useful in an interacting quantum field theory.

### Adding interactions

The quantum formalism was so far developed for the harmonic oscillator (which corresponds to the modes of a free 4-derivative quantum field theory), finding that the quantum theory has a positive-energy spectrum and no runaway behaviors. Adding interactions, the quantum interacting inherits all these good properties, as long as interactions are perturbative and as long as the interacting Hamiltonian *H* remains self-adjoint.

The second issue was the main obstacle to past attempts of adding ad hoc unusual *i* factors in order to make the quantum free theory consistent [33–42] (normalizable wavefunctions and unitary evolution with negative norm and positive energy eigenvalues): adding extra complex factors can render interactions complex, ruining the theory [43, 44].

In our approach the only extra *i* factor arose from a principal reason: $\dot{q}$ is a *T*-odd coordinate that follows the negative-norm Dirac–Pauli representation. This satisfies all the properties of quantum mechanics, as generalized to negative norms: $\dot{q}$ itself is self-adjoint, like *q* and $\ddot{q}$. Thereby any interaction which is a real function of them is self-adjoint. Our procedure immediately generalizes to the interacting case (in agravity [12] all interactions are dictated by general covariance).

The perturbativity assumption means that, as long as the energy spectrum of the free oscillator gets slightly distorted by interactions, the energy eigenvalues will remain real and bounded from below (strongly interacting theories could also be good; however, they seem not needed for the physical application to agravity [12]).

One might worry that, even if all energy eigenvalues are positive, the theory possesses negative-norm states with $\langle \psi | H|\psi \rangle <0$. Equation (29) shows how transition amplitudes can be computed trough perturbation theory: we see that the energy eigenvalues are the quantity that enters into the conservation of energy. Thereby a theory where all eigenvalues of *H* (of $H_0$ in the perturbative expansion) are positive is consistent. As usual, perturbative computations can be systematized in terms of the propagator. By expressing $q=q_1$ in terms of the annihilation and creation operators $a_{i}$, $a_{i}^\dagger$ through Eqs. (11) and (12) and using the commutation relations $[\tilde{a}_i, \tilde{a}_i^\dagger ]=s_i$ we find the propagator

73a $$\begin{aligned}&\langle 0 | T q(t)q (t')|0\rangle \nonumber \\&\quad = \langle 0 | \theta (t-t') q(t)q(t')+\theta (t'-t)q(t')q(t) |0\rangle \end{aligned}$$

73b $$\begin{aligned}&\quad =\frac{1}{\omega _1^2-\omega _2^2} \sum _i \frac{s_i}{2\omega _i} [\mathrm{e}^{i \omega _i (t-t')}\theta (t'-t)\nonumber \\&\qquad +\,\mathrm{e}^{i\omega _i (t'-t)}\theta (t-t')] \end{aligned}$$

73c $$\begin{aligned}&\quad =i \frac{1}{\omega _1^2-\omega _2^2} \int \frac{\mathrm{d}E}{2\pi } \sum _i \frac{s_i \, \mathrm{e}^{-iE(t-t')}}{E^2-\omega _i^2+i\epsilon }\end{aligned}$$

73d $$\begin{aligned}&\quad =\int \frac{\mathrm{d}E}{2\pi } \frac{-i\,\mathrm{e}^{-iE(t-t')}}{(E^2-\omega _1^2+i\epsilon )(E^2-\omega _2^2+i\epsilon )}, \end{aligned}$$

where $\epsilon$ is a small positive quantity and we used $s_1=-1$ and $s_2=1$ in the last step.

One might worry that, using the Heisenberg picture, operators satisfy the time evolution equation $\dot{A} = -i [A,H]$, which looks dangerously similar to the classical equation of motion, as given by Poisson parentheses, which has runaway solutions. However, the quantum solutions are equal to the classical solutions only in a free theory. In general operators are not numbers, and the difference (in particular, the Dirac–Pauli representation) manifests when non-linear interactions are present. As is well known, the Heisenberg equations are in general solved by $A(t) =U^\dagger (t) A(0) U(t)$. So, all good properties of negative-norm states found in the Schrödinger picture remain valid in the Heisenberg picture, given that they are equivalent.

### Extension to quantum field theory

As is well known, a single harmonic-oscillator degree of freedom *q*(*t*) is the building block for a field such as $\phi (t,x,y,z)$ or $g_{\mu \nu }(t,x,y,z)$. The expansion of a field in Fourier modes with given momentum works in the 4-derivative case similarly to the 2-derivative case. As long as, at the end, we are only interested in *S*-matrix elements, all the detailed structure of the quantum mechanical theory, such as the wavefunctions, gets hidden behind the commutation relations of Eq. (13), which hold separately for each mode. The usual $i \epsilon$ prescription for the field propagator dictates that amplitudes can be analytically continued from the Euclidean case. Details will be presented elsewhere.

One would like to claim that quantum field theory inherits all good properties of quantum mechanics also when negative norms are present. However, while in quantum mechanics interactions can easily satisfy the condition that avoids ‘tachyonic ghosts’ (namely, the interaction strength between two opposite-norm states must be smaller than their energy difference as discussed in Sect. 3.2), any interesting quantum field theory leads to situations that might violate this condition. The simplest situation where this occurs is the decay of a ghost (for example a massive spin 2 graviton at rest), which can be degenerate with a multi-particle state (for example two photons going in opposite directions with energy equal to half of the ghost mass), such that the interaction, no matter how small, can be smaller than the energy difference. Actually, the ghost is degenerate with an infinite number of similar states, such that an appropriate limit procedure is needed: in the positive-norm case, entropic considerations allow to interpret this situation as particle decay. A 4-derivative kinetic term $\Pi (p) =-( p^2 - m_1^2)(p^2-m_2^2)$ acquires a positive imaginary part. We will explore if ‘ghost decay’ can be interpreted like in [32].

### Ghost does not play dice?

So far we carefully avoided talking about probabilities.

The theory is unitary in a negative-norm space. Thereby the only remaining difficulty is assigning an interpretation to states that entangle positive-norm components with negative-norm components. The Copenhagen interpretation added an extra ingredient external to the deterministic formalism of quantum mechanics: the Born postulate, according to which:

“when an observable corresponding to a self-adjoint operator *A* is measured in a state $| \psi \rangle$, the result is an eigenvalue $A_n$ of *A* with probability

74 $$\begin{aligned} P_n=\frac{\langle \psi | \Pi _n|\psi \rangle }{\langle \psi | \psi \rangle }\quad \hbox {where}\ \Pi _n = \frac{| n \rangle \langle n |}{\langle n | n \rangle } \end{aligned}$$

is the projector over the eigenstate $| n \rangle$ of *A*”.

For positive norms, these $P_n$ satisfy the probability rules $0\le P_n\le 1$ and $\sum _n P_n=1$; the average value of *A* satisfies $\langle \psi | A|\psi \rangle /\langle \psi | \psi \rangle = \sum _n A_n P_n$.

At the moment we do not have a satisfactory generalization to indefinite norm. Even worse, the Born postulate is unsatisfactory by itself, given that it describes a non-local collapse of the wavefunction [53–55]. In order to make progress, one needs to operate close to the heart of quantum mechanics. As is well known this presents fatal risks: physicists tend to become philosophers. We conclude by listing some interpretations of quantum mechanics, equivalent to the Copenhagen interpretation, which could lead to a satisfactory indefinitive-norm quantum mechanics.

1. Feynman clarified the ontological basis of the Born postulate: it agrees with experiments, so ‘shut up and compute’. All experiments have so far been performed with positive-norm states. The negative-norm graviton predicted by agravity is beyond the reach of present experiments. On the one hand, this is good because it means that Einstein’s general relativity is recovered at large distances; on the other hand, however, we do not have experimental guidance. Lee and Wick proposed that the interpretation issue is bypassed, given that in quantum field theory we can only observe asymptotic states, which are made of positive-norm quanta [22, 23]. The Lee–Wick idea may be applied to the gravitational theory proposed by Stelle [2], as discussed in [6, 56].
2. Any self-adjoint Hamiltonian *H* gives unitary evolution with respect to many different norms, since each energy eigenstate evolves picking just a phase. Defining ghost parity $\mathcal{G}$ to be the metrics in the special basis of energy eigenstates and $| ^\psi \rangle = \mathcal{G}^{-1} | _\psi \rangle$, a possible generalization of the Born postulate to generic norm is (see also [33–42])
  75 $$\begin{aligned} P_n = \langle ^\psi | \Pi _n|_\psi \rangle \quad \hbox {where}\ \Pi _n = | ^n \rangle \langle _n |. \end{aligned}$$
  The example of Sect. 3.2 gets converted into normal oscillations with mixing angle $\sin ^22\theta =E_I^2/E_R^2$. However, $\langle _\psi | A|_\psi \rangle$ is real but does not have a probabilistic interpretation, while $\langle ^\psi | A|_\psi \rangle$ has a probabilistic interpretation but can be complex.
3. Various authors claim that the Born postulate is just an emergent phenomenon (somehow like friction) that follows from the fundamental deterministic equations when applied to complex systems in view of spontaneous decoherence [57–59].
4. Cramer [60, 61] proposed a “transactional interpretation”, claiming that EPR non-locality results from a cancellation of advanced and retarded waves, in a time-symmetric set-up (see also [62]) inspired by the analogous formulation of classical electro-dynamics proposed by Dirac and Feynman–Wheeler. The $\langle \psi ' | \psi \rangle$ amplitude in the Dirac–Pauli coordinate representation supports the interpretation as being the overlap of a wave $\psi$ moving forward in time with a wave $\psi '$ moving backwards in time.

We plan to further investigate such issues.

## Conclusions (so far)

We presented the quantization of 4-derivative theories, finding that a unique structure emerges. We can summarize it as follows.

Quantum mechanics has its usual visible face, where a coordinate operator *q* is represented as $q| x \rangle =x| x \rangle$. But quantum mechanics also has a hidden face, where $q| x \rangle =ix| x \rangle$, as first pointed out by Dirac and Pauli. Both *q* and *p* of a canonical pair $[q,p]=i$ are self-adjoint in both representations. The main difference is that the usual representation implies positive norms and *q* is naturally even under time reflection *T*, while the DP representation leads to states with indefinite norm and to a naturally *T*-odd *q* (in view of the *i* factor and of the fact that *T* is anti-unitary).

The Ostrogradski formulation of a 4-derivative degree of freedom *q*(*t*) (summarized in Sect. 2) employs two canonical coordinates: $q_1=q$ and $q_2 = \dot{q}$. For the first time we have observed that $q_1$, which is *T*-even, naturally follows the usual representation, while $q_2$, which is *T*-odd, naturally follows the Dirac–Pauli negative-norm quantization. This leads to a sensible quantum theory with positive energies and normalizable wavefunctions, as discussed in Sect. 4.

In Sect. 3 we presented a new formalism appropriate for generic-norm quantum mechanics, introducing ‘covariant’ $| _n \rangle$ and ‘contravariant’ $| ^n \rangle$ basis states. This clarifies why a self-adjoint linear operator can be represented by a matrix that, in some basis, is not hermitian. A self-adjoint Hamiltonian leads to unitary time evolution, in the sense that the negative norm is preserved. Given that $q,\dot{q},\ddot{q}, \ldots$ are self-adjoint, a Hamiltonian which is a generic real function of them is self-adjoint, leading to sensible interacting quantum theory provided that one avoids tachyons, an observation that was previously overlooked. The usual condition that the theory should be free of tachyons is generalized to negative-norm quantum mechanics.

In Sect. 5 we presented the path integral formulation of negative-norm quantum mechanics. The classical Hamiltonian becomes complex. Another new result of this paper is the proof that the normalizable wavefunctions found in the operator formalism are recovered from the path integral after performing naive manipulations over ill-defined objects and/or analytic continuations. In particular, the version of the path integral in Euclidean time $t_E = it$ is well defined, and reproduces the usual wavefunctions taking into account that $\mathrm{d}q/\mathrm{d}t_E$ already coincides with the Dirac–Pauli $\dot{q}$.

The fact that (1) our approach leads to normalizable wavefunctions and (2) these wavefunctions can also be deduced from a well-defined Euclidean path integral clearly show that the right quantization for $\dot{q}$ is the Dirac–Pauli one.

Two issues must be addressed before these results can be used to obtain a predictive renormalizable quantum theory of gravity: generalization to quantum field theory, and generalization of the Born probabilistic interpretation to negative norms.

## Footnotes

1 Unlike in the case of the hydrogen atom emphasized by Woodard [16, 17], where the instability eliminated by quantum mechanics occurs only in one point of the configuration space.

2 Here *T* denotes the matrix transpose; $*$ denotes complex conjugation; $\dagger$ denotes the adjoint operation, which generalizes the usual hermitian conjugation and reduces to it in the positive-norm case. We never use $\dagger$ to denote hermitian conjugation of a matrix.

3 Alternative routes lead to the same conclusion. For example, one can use the *T*-even $\ddot{q}$ (instead of $\dot{q}$) as second canonical coordinate. In the Ostrogradski formalism $\ddot{q} = - p_2$ is a momentum. So again one gets a canonical coordinate with unusual *T*-parity (normally a momentum is *T*-odd). In general, the invariance of the commutation relation $[q_2,p_2]=i$ under the anti-unitary time-inversion implies that $q_2$ and $p_2$ have opposite *T*-parities. One can switch $q_2\leftrightarrow p_2$ in order to restore their usual *T*-parities: but their commutator changes sign, implying again negative-norm quantization. This is indeed what happens in the auxiliary variable formalism, used in various forms in the literature as an alternative to the canonical Ostrogradski formalism (see e.g. [28, 29, 32, 49]). This formalism is convenient when dealing with quantum field theory instead of quantum mechanics with a finite number of degrees of freedom. In order to facilitate the contact, we summarize the auxiliary variable formalism below. Restarting from the Lagrangian in Eq. (3), we add zero as a perfect square containing an auxiliary variable *a*:

47 $$\begin{aligned} {\mathscr {L}} = {\mathscr {L}}+ \frac{1}{2} \bigg [ \ddot{q} + (\omega _1^2+\omega _2^2)\frac{q}{2} - \frac{a}{2}\bigg ]^2. \end{aligned}$$

Expanding the square cancels both the second-order and the fourth-order kinetic terms for *q* leaving

48 $$\begin{aligned} {\mathscr {L}} =-\frac{a \ddot{q}}{2} + (\omega _1^2-\omega _2^2)^2\frac{q^2}{8} - (\omega _1^2+\omega _2^2)\frac{aq}{4} + \frac{a^2}{8}-V(q). \end{aligned}$$

Going to the free theory $V=0$, we can diagonalize the kinetic and mass term through the field redefinition

49 $$\begin{aligned}&\left\{ \begin{array}{l} a = \sqrt{\omega _1^2 - \omega _2^2}(\tilde{q}_2 - \tilde{q}_1)\\ q = (\tilde{q}_2 + \tilde{q}_1)/{\sqrt{\omega _1^2-\omega _2^2}} \end{array}\right. \qquad \hbox {i.e.}\nonumber \\&\qquad \tilde{q}_{1,2} =\frac{q}{2} \sqrt{\omega _1^2-\omega _2^2} \mp \frac{a}{2\sqrt{\omega _1^2-\omega _2^2}} \end{aligned}$$

obtaining two decoupled oscillators,

50 $$\begin{aligned} {\mathscr {L}} = \frac{\dot{\tilde{q}}_2^2 - \omega _2^2 \tilde{q}_2^2}{2} - \frac{\dot{\tilde{q}}_1^2 - \omega _1^2 \tilde{q}_1^2}{2} . \end{aligned}$$

From its classical solution, $a = 2 \ddot{q} +(\omega _1^2+\omega _2^2)q$, we see that *a* roughly corresponds to the Ostrogradski $p_2$. Furthermore, inserting such classical solution in Eq. (49) one recovers the formalism used in [28, 29]

51 $$\begin{aligned} \tilde{q}_2 = \frac{\ddot{q}+\omega _1^2 q}{\sqrt{\omega _1^2-\omega _2^2}},\quad \tilde{q}_1 = - \frac{\ddot{q}+\omega _2^2q}{\sqrt{\omega _1^2-\omega _2^2}}. \end{aligned}$$

4 The possibility of converting a non-normalizable wavefunction $\psi _0 \propto \mathrm{e}^{z^2/2}$ into a normalizable one by restricting $z=x+iy$ to the imaginary axis, rather than along the real axis, was presented as an ad hoc recipe to get something sensible in earlier works by Bender and Mannheim [33–42]. At the technical level, their approach differs from ours because they added an *i* factor to the variable *q*, rather than to $\dot{q}$. Our approach follows from general considerations, and it has the advantage that *q*, *p* and thereby the Hamiltonian are self-adjoint (although their matrix representations look anti-hermitian), such that the generalization to an interacting theory will be immediate (Sect. 6.1).

5 In the limit $\mathrm{d}t = t_f- t_i\rightarrow 0$ the classical action becomes

63 $$\begin{aligned} S_\mathrm{cl} \simeq \frac{6}{\mathrm{d}t^3}\left( q_f - q_i - \mathrm{d}t \frac{\dot{q}_i + \dot{q}_f}{2}\right) ^2 + \frac{(\dot{q}_f - \dot{q}_i)^2}{2 \, \mathrm{d}t}+\cdots \end{aligned}$$

which is minimal for a motion with constant speed $(\dot{q}_i + \dot{q}_f)/2$. The classical action satisfies $S(q_f,\dot{q}_f, t_f ; q_i ,\dot{q}_i, t_i) = - S( q_i ,\dot{q}_i, t_i ;q_f,\dot{q}_f, t_f )$ as well as $S_\mathrm{cl}(q_f,\dot{q}_f,t_f; q_i,\dot{q}_i, t_i)=-S_\mathrm{cl}(q_f,-\dot{q}_f,t_i; q_f,-\dot{q}_i, t_i)$.

6 The classical Hamiltonian is real if one instead uses the equivalent oscillator basis $\tilde{q}_1,\tilde{q}_2,\tilde{p_1},\tilde{p}_2$ of Eq. (53).

7 Hawking and Hertog [28, 29] found a non-normalizable Minkowskian wavefunction because they expressed Eq. (72) in terms of $\dot{q}$, which is not the canonical coordinate $q_2$ appropriate for 4-derivative theories. Their proposal of integrating out $\dot{q}$ in the Euclidean before performing the analytic continuation to the Minkowskian is not necessary: the Minkowskian wavefunctions are normalizable if the appropriate analytic continuation is performed.
